# Discrepancies between media portrayals and actual demographics of eating disorders in TV and film: implications of representation

**DOI:** 10.1186/s40337-023-00892-y

**Published:** 2023-09-18

**Authors:** Lucy Bassett, Maya Ewart

**Affiliations:** 1 Batten School of Leadership and Public Policy, University of Virginia, Charlottesville, VA USA; 2 New York City Human Resources Administration, New York, NY USA

**Keywords:** Eating disorder, Anorexia nervosa, Bulimia nervosa, Media, Film, Movies, TV, Gender, Age, LGBTQ+

## Abstract

**Background:**

Media has a reputation for painting a narrow, simplistic, sensationalized portrait of eating disorders. There is little analysis of how film and TV portray eating disorders nor the implications of this representation. This study fills that gap by comparing demographics of US film and TV characters since the 1980s to actual population demographics of people with eating disorders.

**Methods:**

We compiled a dataset of TV and movie characters with eating disorders and categorized characters’ gender, age, race/ethnicity, and sexual orientation. We narrowed the dataset to include only US media depictions to facilitate comparisons with empirical prevalence, resulting in a total of 66 characters over the period 1981 to 2022. We then compared the demographic characteristics of our sample to national statistics on eating disorder prevalence.

**Results:**

US media depictions of eating disorders overrepresented characters who were heterosexual (75.56%), White (84.85%), women (89.39%), and under age 30 (84.85%). This does not accurately reflect the populations experiencing eating disorders in the US.

**Conclusions:**

Eating disorders have an image problem. TV and movies inaccurately portray them as primarily affecting heterosexual, White, women under age 30. Misrepresentation could fuel existing stigmas that inhibit individuals with eating disorders from seeking and receiving treatment. It could also perpetuate stereotypes that fuel misperceptions of the disease by medical providers, families, and policymakers. We recommend more accurate representation in the media to better reflect current demographics and increase awareness of the range of people who can experience eating disorders.

## Media and eating disorders

Media has a reputation for painting narrow, simplistic, and sensationalized portraits of eating disorders [1], presenting them as affecting “skinny, White, affluent girls” (the SWAG stereotype) [2]. Scholars have found that representation in film can influence viewers’ attitudes and behaviors. Indeed, film often reiterates existing stereotypes that can lead viewers to use these portrayals as a frame to view and understand people [3].

There is little research on eating disorder representation in the media beyond social media or news coverage [4]. Exceptions to this include a general scan of eating disorder depictions in film [5] and a review focusing almost exclusively on race [6]. Representation on TV has been largely overlooked. This analysis seeks to fill that gap by assessing the degree to which TV and film depictions of eating disorders reflect the actual demographics in the US, looking at gender, age, race, and sexual orientation.

## Methodology

We conducted Boolean searches of key terms using Google and Google Scholar search engines. Terms included “TV,” “movie,” “film,” “character,” “eating disorder,” “anorexia nervosa,” “bulimia nervosa,” “binge eating disorder,” “orthorexia,” and “media representation.” From these searches and existing lists [7, 8], we created a dataset of 96 TV and movie characters with eating disorders.

We did not include documentaries; all characters were portrayed by actors. We included depictions of anorexia nervosa, binge eating disorder, bulimia nervosa, orthorexia nervosa, and unspecified disorders. Disorders were deemed “unspecified” if a character exhibited eating disorder symptomatology (e.g., restriction, purging) but their condition was neither explicitly mentioned in the film or TV show nor noted in any commentary as being a specific disorder. The sample consisted of movies and TV from 1981 to 2022.

We then created a demographic categorization for each character including their gender, age, race/ethnicity, and sexual orientation. Coding focused on the way the character was depicted in the film or TV show (story), not the actor’s demographic characteristics. Multiple raters coded the dataset, and the final categorization reflects the consensus reached. Gender and race/ethnicity were coded based on how the character was identified in the story and/or documentation describing the character. If age was not specified in the story or any related documentation, we used the age of the actor at the time of filming. Characters were coded as heterosexual if all their romantic partners in the story were of the opposite sex. If a character had no romantic partners or plot lines, their sexual orientation was coded as “unspecified.”

To facilitate demographic comparisons, we narrowed the dataset to include only US media depictions, resulting in a total of 66 characters. We then compared the demographic breakdowns of our sample to national statistics on eating disorder prevalence.

## Limitations

Characters may have been missed or imperfectly categorized given not all demographics are made explicitly clear in TV and movies. We did not analyze physical size, socio-economic status, or eating disorder representation outside the US. Further research is needed to explore media portrayal of eating disorders within diverse cultural and country contexts.

## Results

The most common eating disorder in the dataset was anorexia nervosa (48.48%, n = 32), followed by bulimia nervosa (39.39%, n = 26.) (All diagnoses are shown on Table 1.) The majority of characters were White (84.85%, n = 56); 9.09% (n = 6) were Latin American, and 6.06% (n = 4) were Black. No character was Asian American Pacific Islander (AAPI) or Indigenous. The majority of characters were women (89.9%, n = 59), 10.61% were men (n = 7) and 6.06% (n = 4) were aged 40 or over. The majority of characters were heterosexual (75.76%, n = 50) or their sexuality was unspecified (21.21%, n = 14). Two characters identified as bisexual, pansexual or lesbian (4.55%) and all were cisgender.

**Table 1:** Eating disorder diagnoses of characters

	Anorexia nervosa	Bulimia nervosa	Binge-eating disorder	Orthorexia	Unspecified
Number	32	26	7	1	9
Percent	48.48%	39.39%	10.61%	1.52%	13.64%

The total numbers by eating disorder exceed the total number of characters because some characters had more than one type of eating disorder

## Discussion

Overall, we found that recent media representation does not accurately reflect the US population of people with eating disorders. The majority of characters in the dataset were heterosexual, White females under age 30. This is a misrepresentation of the demographics of individuals in the US with eating disorders in terms of gender, age, race/ethnicity, and sexual orientation.

Just 10.61% of the characters were men. Yet approximately one third of people in the US with an eating disorder are male and 1 in 7 males will experience an eating disorder by age 40 [9–11]. Some experts assume the number is much higher because many men do not identify their condition [12].

Eating disorders are not limited to young people. In a 2021 study of 1,849 women over 50 in the US, an estimated 13% had eating disorder symptoms [13]. While the peak age for anorexia nervosa is estimated to be 26, for disorders like bulimia nervosa and binge eating disorder, peak ages are estimated at 47 and 70 [14]. However, only 6% of characters were over 40.

Black, Indigenous, People of Color (BIPOC) individuals were notably underrepresented in our dataset. Over 80% of characters were White. However, compared to White peers, Black adolescents in the US are 50% more likely to engage in bulimic behaviors [15]. There were 20 times more White characters with bulimia nervosa than Latin American characters, yet Latin American and White Americans exhibit similar rates of bulimia nervosa [16]. We found no media portrayals of Indigenous or AAPI individuals with eating disorders.

The LGBTQ+ community was also extremely underrepresented in our sample. Less than 5% of characters were LGBTQ+ and none were trans. However, compared to heterosexual and cisgender counterparts, LGBTQ+ adults and adolescents have a higher incidence of both eating disorders and disordered eating behaviors [17].

Looking at trends over time, we found that media depictions of eating disorders have evolved slightly, but still do not accurately reflect empirical evidence. In our review, no male characters with eating disorders appeared in films until 2014. There were no BIPOC characters with eating disorders until 2000.

Lack of representation in US TV and film depictions of eating disorders paints a false portrait that only young White women have eating disorders. This media misrepresentation is problematic for several reasons, with implications for recognition of the disease, diagnosis, and treatment [2].

Eating disorders are associated with considerable stigma; those who are affected often hide their experience or fail to seek help. This contributes to limited visibility and societal awareness of eating disorders [18], especially for groups that do not fit eating disorder stereotypes. For example, men with eating disorders are less likely to seek treatment and are often “under-diagnosed, undertreated, and misunderstood” by service providers [11, 12]. For individuals to receive the treatment and support they need, they must first recognize that they *can* have an eating disorder.

Diagnosis is often based on misperceptions of eating disorders, resulting in important gaps. Diagnostic tools to identify eating disorders are typically designed for women [19]. Although Black individuals often experience anorexia nervosa for longer periods of time than White individuals, they are less likely to be diagnosed [20]. The same diagnosis gap is evidenced for older people, who are less likely to be diagnosed with eating disorders, even though they tend to experience more severe health consequences [21].

In eating disorder treatment programs, women with eating disorders greatly outnumber male counterparts. Men with eating disorders often feel uncomfortable opening up in a female-dominated environment, especially when group discussion primarily focuses on female symptomatology [22]. The same is true for BIPOC people with eating disorders, who often feel marginalized in treatment environments [23, 24].

## Recommendations

We recommend the media commit to showing a more diverse array of people affected by eating disorders [1]. Organizations like Mindframe (Australia) and Beat (UK) have prepared guidelines for media reporting on eating disorders [25]. The Hollywood, Health & Society program supports the entertainment industry through resources, consultations, and panel discussions with experts and “real people” with relevant lived experience [26]. Many TV programs have already benefited from this support on topics such as abortion, HIV/AIDS, and opioid addiction. Eating disorders should be next.

## Conclusion

Eating disorders have an image problem in the media today. Accurate representation of eating disorders would show providers, survivors, and the general public that anyone can have an eating disorder. This broader understanding can destigmatize eating disorders and open the door for more individuals to receive support and care.

## Data Availability

The datasets used and analyzed during the current study are available from the corresponding author on reasonable request.

## Abbreviations

AAPI: Asian American and Pacific islander
BED: Binge-eating disorder
BIPOC: Black, indigenous, people of color
LGBTQ+: Lesbian, gay, bisexual, transgender, queer and/or questioning
SWAG: Skinny, white, affluent girls
